# Supplementary material for: Genome-wide sequencing-based identification of methylation quantitative trait loci and their role in schizophrenia risk
Source: Nat Commun. 2021 Sep 2;12:5251. doi: 10.1038/s41467-021-25517-3 (PMC8413445; doi:10.1038/s41467-021-25517-3)
Supplement: Supplementary file 1 — Supplementary Information [file 41467_2021_25517_MOESM1_ESM.pdf]

Title: Genome-wide sequencing-based identification of methylation quantitative trait loci and their role in schizophrenia risk

Authors:

Kira A. Perzel Mandell[1,2], Nicholas J. Eagles[1], Richard Wilton[3], Amanda J. Price[1,2], Stephen A. Semick[1], Leonardo Collado-Torres[1], William S. Ulrich[1], Ran Tao[1], Shizhong Han[1,4], Alexander S. Szalay[3,5], Thomas M. Hyde[1,4,6], Joel E. Kleinman[1,4], Daniel R. Weinberger[1,2,4,6,7+], Andrew E. Jaffe[1,2,4,7,8,9,*]

1. Lieber Institute for Brain Development, Johns Hopkins Medical Campus, Baltimore, MD 21205, USA
2. Department of Genetic Medicine, Johns Hopkins University School of Medicine (JHSOM), Baltimore, MD 21205, USA
3. Department of Physics and Astronomy, Johns Hopkins University, Baltimore, MD, USA
4. Department of Psychiatry and Behavioral Sciences, JHSOM, Baltimore, MD, USA
5. Department of Computer Science, JHSOM, Baltimore, MD, USA
6. Department of Neurology, JHSOM, Baltimore, MD, USA
7. Department of Neuroscience, JHSOM, Baltimore, MD, USA.
8. Department of Mental Health, Johns Hopkins Bloomberg School of Public Health (JHBSPH), MD 21205, USA
9. Department of Biostatistics, JHBSPH, Baltimore, MD, USA.

+ drweinberger@libd.org; *andrew.jaffe@libd.org

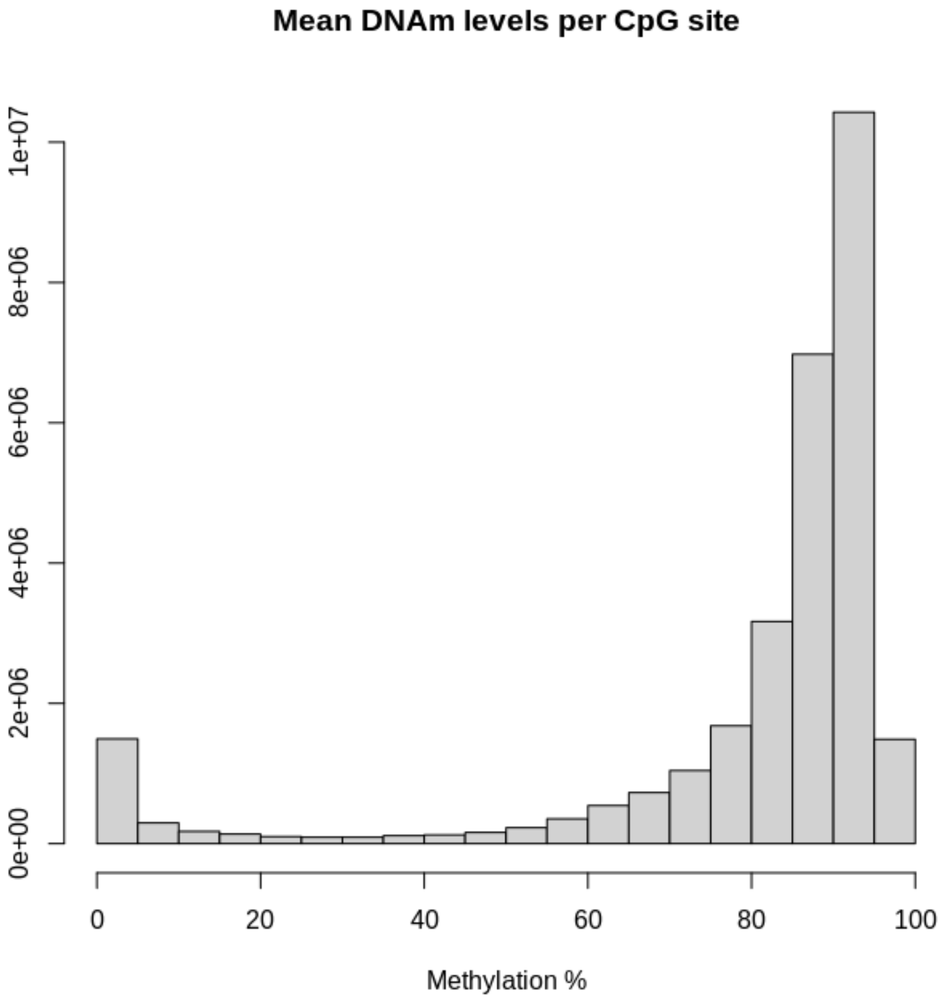

Figure S1: Distribution of DNAm levels across all sites. For each CpG site, the mean DNAm level across all samples was calculated, showing that most CpGs are highly methylated.

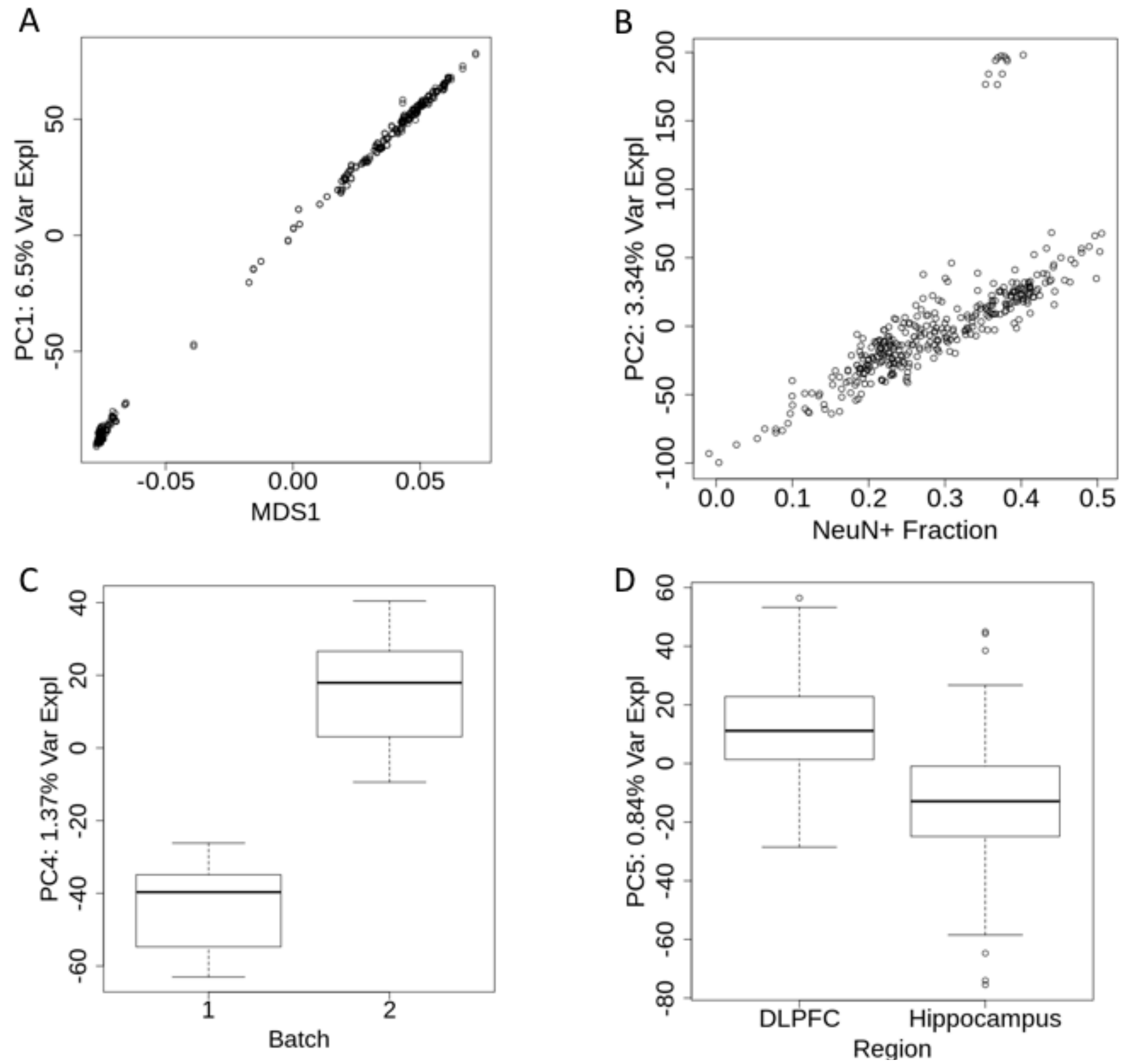

Figure S2: PCA on raw methylation values, excluding sex chromosomes. Principal components of variance are plotted against their most associated factor. **(A)** The top component of variance in raw methylation data corresponds with the top multidimensional scaling (MDS1) component of the genotype data, which is a representation of ethnicity. **(B)** The second principal component aligns with estimated neuronal fraction. Here we also see 11 DLPFC samples with unexplained variance, so they are dropped from analysis. **(C)** The third principal component strongly associates with processing batch. **(D)** The fourth principal component correlates with brain region. n = 344 biologically independent samples.

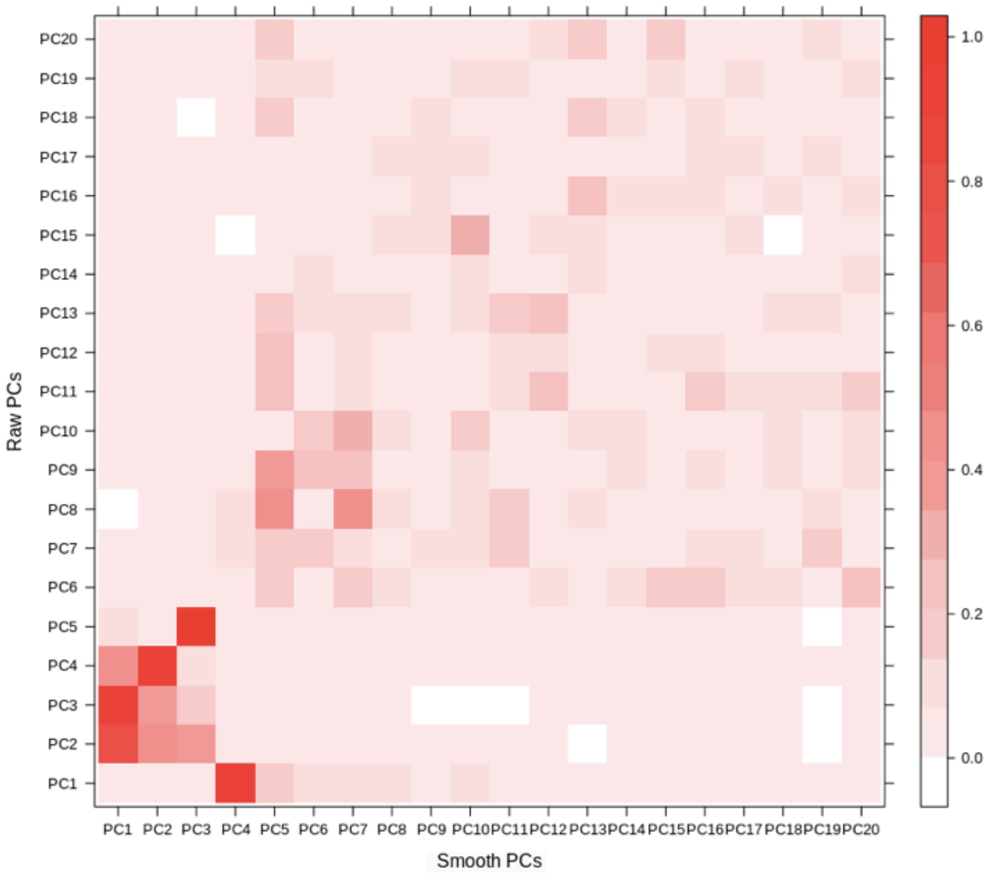

Figure S3: Comparison between top 20 PCs of raw and smooth methylation data. We see that smoothing methylation data alters the principal components of variants, mitigating the effects of ethnicity and increasing the effects of batch and cell composition.

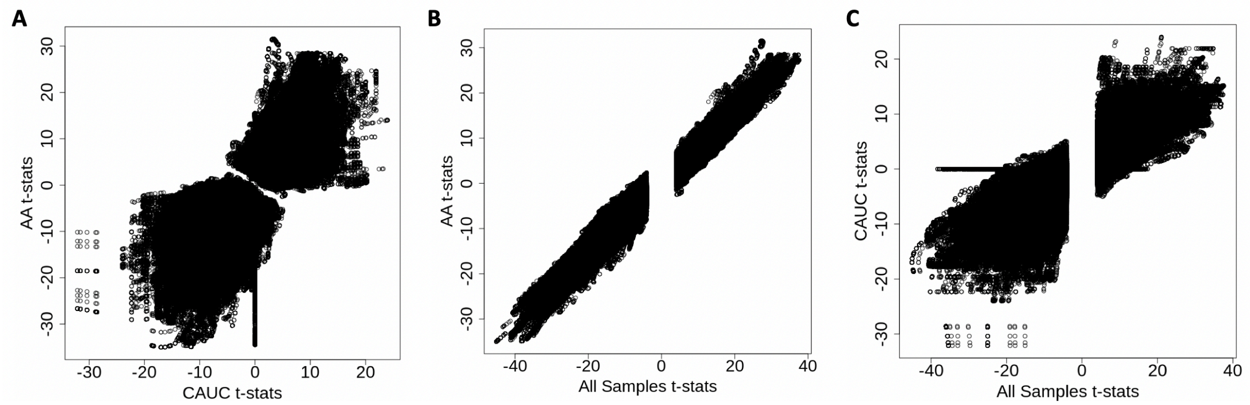

Figure S4: Ethnicity Differences in meQTL t-statistics. For meQTLs identified on chromosome 1, we ran post-hoc meQTL analysis on the samples divided by self-reported ethnicity. **(A)** There are some differences between meQTL strengths between ethnicities, but they are largely correlated and directionally consistent. **(B)** meQTL statistics in AA only were very similar to the overall findings, likely because the majority of our samples were AA. **(C)** CAUC meQTL statistics compared to t-statistics from all samples.

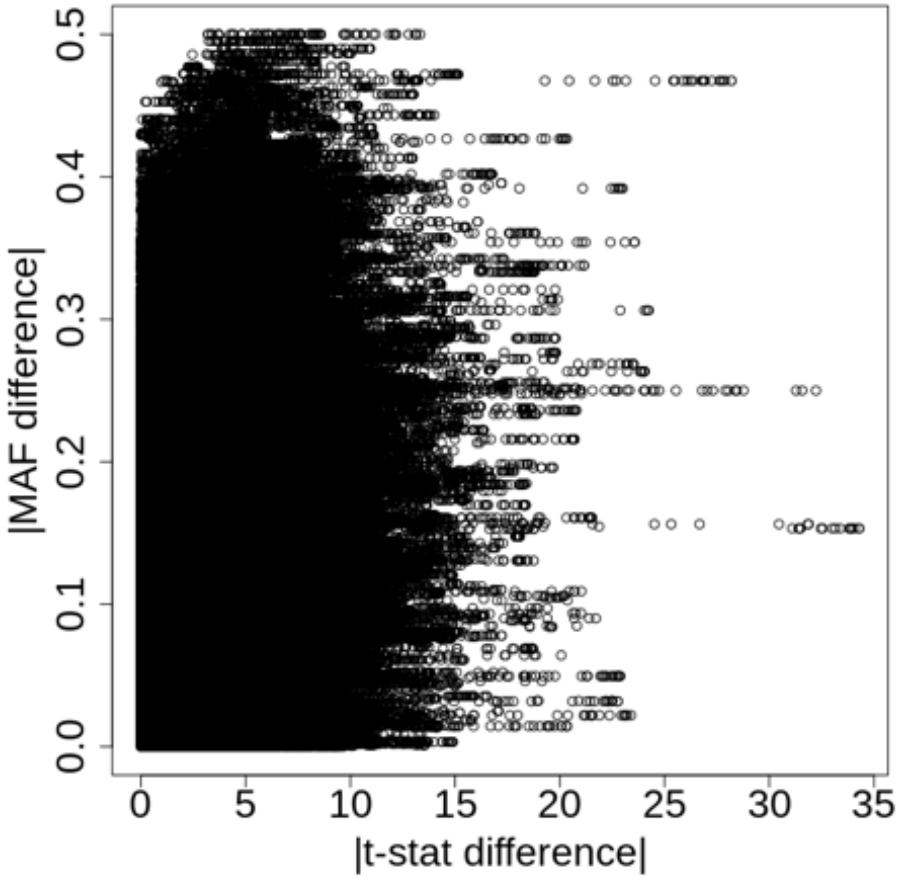

Figure S5: **Lack of association between population MAF differences and meQTL strength.** Though some differences in meQTL strength can be seen between ethnicities, meQTL strength does not associate with SNP MAF or MAF differences between ethnic populations.

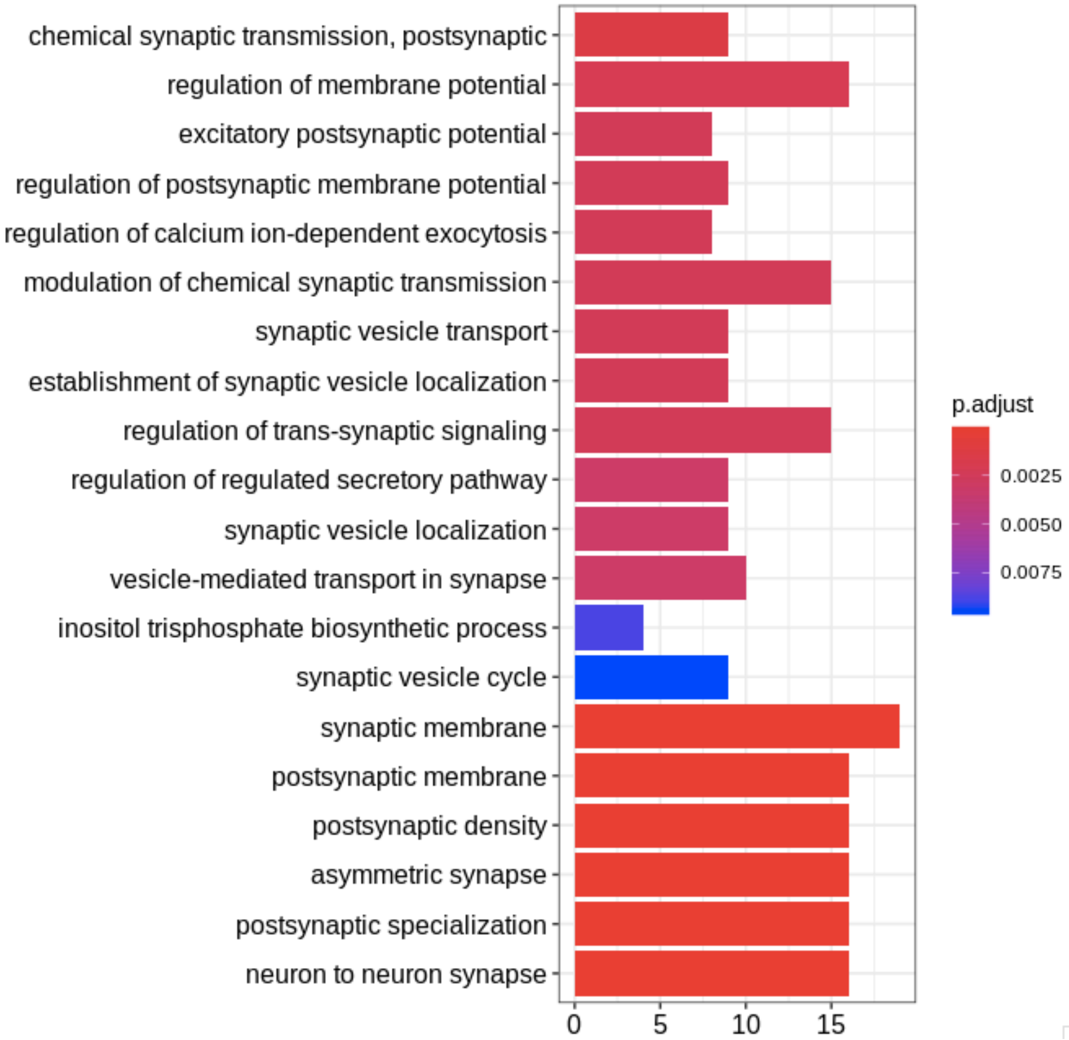

Figure S6: GO Enrichment of schizophrenia risk associated DMRs. We find that DMRs associated with schizophrenia risk variants are in or near genes enriched for synapse related GO terms.

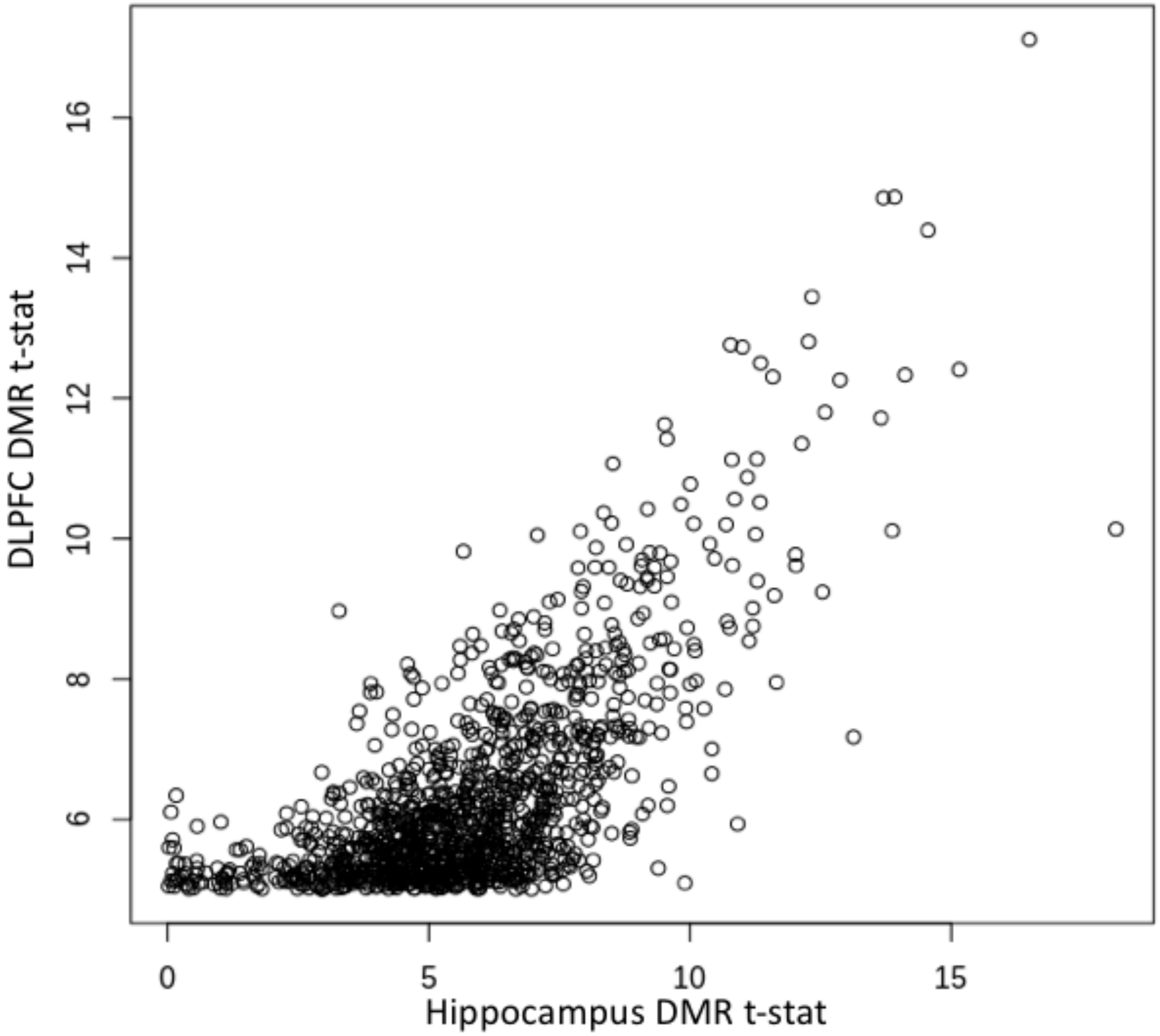

Figure S7: Comparison of Hippocampus association statistics to the statistics of DMRs identified in DLPFC. meQTL association statistics are highly correlated between brain regions.

Supplementary Datasets

Supplementary Dataset 1: Sample Demographic Data

Supplementary Dataset 2: DLPFC CpG meQTLs to SCZD risk SNPs. snps: SNP identifier; cpg: location of CpG meQTL site (hg38); statistic: t-statistic for meQTL association between SNP and CpG; pvalue: P-value for meQTL association; FDR: Benjamin-Hochberg corrected p-value; beta: regression coefficient, or the change in DNAm levels per minor allele copy; snpChr: chromosome of genome where the SNP lies (hg38); snpPos: position on the chromosome where the SNP lies (hg38); snpRsNum: rs number of the genetic variant; snpCounted:the counted/minor allele of the meQTL variant (`beta` is relative to this); snpAlt: the reference allele for the variant; disruptCpG: whether the SNP disrupts any CpG dinucleotides (even one different than the meQTL); methChr: chromosome where the CpG lies (hg38); methPos: position where the CpG lies (hg38); distMethToSnp: genomic distance between SNP and CpG.

Supplementary Dataset 3: HIPPO CpG meQTLs to SCZD risk SNPs. See Table S2 for field descriptions.

Supplementary Dataset 4: DLPFC DMRs formed by SCZD "index" SNPs. chr: chromosome where the differentially methylated region (DMR) lies (hg38); start: start position of the DMR (hg38); end: end position of the DMR (hg38); value: mean meQTL t-statistic within the DMR; area: sum of the meQTL t-statistics within the DMR; cluster, indexStart, and indexEnd are internal indices for rows of the methylation matrix; L: number of CpGs in DMR; clusterL: number of nearby CpGs considered for DMR finding; width: genomic width of DMR; snpPos: position of the schizophrenia index SNP used to find DMR; disruptCpG: whether the SNP disrupts any CpG dinucleotides; distStart: distance of SNP to start of DMR; distEnd: distance of SNP to end of DMR; location: location of DMR relative to genes

Supplementary Dataset 5: HIPPO DMRs formed by SCZD "index" SNPs. See Table S4 for field descriptions.

Supplementary Dataset 6: DLPFC CpH meQTLs to SCZD risk SNPs. See Table S2 for field descriptions.

Supplementary Dataset 7: HIPPO CpH meQTLs to SCZD risk SNPs. See Table S2 for field descriptions.
