# Supplementary material for: Genome-wide sequencing-based identification of methylation quantitative trait loci and their role in schizophrenia risk
Source: Nat Commun. 2021 Sep 2;12:5251. doi: 10.1038/s41467-021-25517-3 (PMC8413445; doi:10.1038/s41467-021-25517-3)
Supplement: Supplementary file 3 — Description of Additional Supplementary Files [file 41467_2021_25517_MOESM3_ESM.docx]

Description of Additional Supplementary Files

| **Results Dataset** | **URL** |
| --- | --- |
| Variance of smoothed DNA methylation levels explained by technical and biological variables across all tested CpGs | <https://mandell-wgbs-meqtl.s3.us-east-2.amazonaws.com/suppData_smooth_variance_explained_annotated.csv.gz> |
| Significant meQTLs across DLPFC using smoothed DNAm values | <https://mandell-wgbs-meqtl.s3.us-east-2.amazonaws.com/suppData_smooth_dlpfc_CpG_meqtls_fdr.csv.gz> |
| Significant meQTLs across HIPPO using smoothed DNAm values | <https://mandell-wgbs-meqtl.s3.us-east-2.amazonaws.com/suppData_smooth_hippo_CpG_meqtls_fdr.csv.gz> |
| Significant CpH-meQTLs across DLPFC | <https://mandell-wgbs-meqtl.s3.us-east-2.amazonaws.com/suppData_smooth_dlpfc_CpH_meqtls_fdr.csv.gz> |
| Significant CpH-meQTLs across HIPPO | <https://mandell-wgbs-meqtl.s3.us-east-2.amazonaws.com/suppData_smooth_hippo_CpH_meqtls_fdr.csv.gz> |
| Associations between smoothed DNAm values and age across the DLPFC for all significant sites | [https://mandell-wgbs-meqtl.s3.us-east-2.amazonaws.com/suppData_smooth_dlpfc_CpG](https://mandell-wgbs-meqtl.s3.us-east-2.amazonaws.com/suppData_smooth_dlpfc_CpG_age.csv.gz)  [_age.csv.gz](https://mandell-wgbs-meqtl.s3.us-east-2.amazonaws.com/suppData_smooth_dlpfc_CpG_age.csv.gz) |
