# Supplementary material for: Genome-wide sequencing-based identification of methylation quantitative trait loci and their role in schizophrenia risk
Source: Nat Commun. 2021 Sep 2;12:5251. doi: 10.1038/s41467-021-25517-3 (PMC8413445; doi:10.1038/s41467-021-25517-3)
Supplement: Supplementary file 11 — Reporting Summary [file 41467_2021_25517_MOESM11_ESM.pdf]

nature portfolio

Corresponding author(s): Andrew E Jaffe

Last updated by author(s): Jul 21, 2021

Reporting Summary

Nature Portfolio wishes to improve the reproducibility of the work that we publish. This form provides structure for consistency and transparency in reporting. For further information on Nature Portfolio policies, see our Editorial Policies and the Editorial Policy Checklist.

Statistics

For all statistical analyses, confirm that the following items are present in the figure legend, table legend, main text, or Methods section.

n/a	Confirmed	
☐	☒	The exact sample size (n) for each experimental group/condition, given as a discrete number and unit of measurement
☐	☒	A statement on whether measurements were taken from distinct samples or whether the same sample was measured repeatedly
☐	☒	The statistical test(s) used AND whether they are one- or two-sided<br>*Only common tests should be described solely by name; describe more complex techniques in the Methods section.*
☐	☒	A description of all covariates tested
☐	☒	A description of any assumptions or corrections, such as tests of normality and adjustment for multiple comparisons
☐	☒	A full description of the statistical parameters including central tendency (e.g. means) or other basic estimates (e.g. regression coefficient) AND variation (e.g. standard deviation) or associated estimates of uncertainty (e.g. confidence intervals)
☐	☒	For null hypothesis testing, the test statistic (e.g. F, t, r) with confidence intervals, effect sizes, degrees of freedom and P value noted<br>*Give P values as exact values whenever suitable.*
☒	☐	For Bayesian analysis, information on the choice of priors and Markov chain Monte Carlo settings
☒	☐	For hierarchical and complex designs, identification of the appropriate level for tests and full reporting of outcomes
☐	☒	Estimates of effect sizes (e.g. Cohen's d, Pearson's r), indicating how they were calculated

Our web collection on statistics for biologists contains articles on many of the points above.

Software and code

Policy information about availability of computer code

Data collection	The raw WGBS data was processed using FastQC to control for quality of reads, Trim Galore to trim reads and remove adapter content, Arioc for alignment to the GRCh38.p12 genome (obtained from ftp://ftp.ncbi.nlm.nih.gov/genomes/all/GCA/000/001/405/GCA_000001405.27_GRCh38.p12/GCA_000001405.27_GRCh38.p12_assembly_structure/Primary_Assembly/assembled_chromosomes/), duplicate alignments were removed with SAMBLASTER, and filtered with samtools (v1.9) to exclude all but primary alignments with a MAPQ >= 5. We used the Bismark methylation extractor to extract methylation data from aligned, filtered reads. We then used the bsseq R/Bioconductor package (v1.18) to process and combine the DNA methylation proportions across the samples for all further manipulation and analysis. After initial data metrics were calculated, the methylation data for each sample was locally smoothed using BSmooth with default parameters for downstream analyses.
Data analysis	We used R package Matrix eQTL (v2.3) in all meQTL analyses. We estimated SNP-heritability of DNAm for each CpG site using the GCTA software. We performed gene ontology and gene set enrichment using clusterProfiler (v3.12). Differential methylation analyses for both diagnosis and age were performed using linear regression modelling, accounting for sex, estimated neuronal fraction, batch, and the top 3 MDS components from genotype data. The regression analyses above were formed using limma (v3.30). LDSC was performed using the LDSC package (v1.0) and accessory analyses included were aided by jaffelab (v0.99.20) and qvalue (v2.20). Analysis code that accompanies this paper is provided on GitHub (https://github.com/LieberInstitute/wgbs_meqtl_analysis) and available at Zenodo (doi: 10.5281/zenodo.5113698)

For manuscripts utilizing custom algorithms or software that are central to the research but not yet described in published literature, software must be made available to editors and reviewers. We strongly encourage code deposition in a community repository (e.g. GitHub). See the Nature Portfolio guidelines for submitting code & software for further information.

Data

Policy information about availability of data

All manuscripts must include a data availability statement. This statement should provide the following information, where applicable:

- Accession codes, unique identifiers, or web links for publicly available datasets
- A description of any restrictions on data availability
- For clinical datasets or third party data, please ensure that the statement adheres to our policy

We have created a user-friendly and fast meQTL browser that allows searching by SNPs or cytosines by genomic regions (chr.start-end) at https://eqtl.brainseq.org/WGBS_meQTL/ . Raw and processed nucleic acid sequencing data generated to support the findings of this study are available via the PsychENCODE Knowledge Portal (https://psychencode.synapse.org/). The PsychENCODE Knowledge Portal is a platform for accessing data, analyses, and tools generated through grants funded by the National Institute of Mental Health (NIMH) PsychENCODE program. Data is available for general research use according to the following requirements for data access and data attribution: (https://psychencode.synapse.org/DataAccess). For access to content described in this manuscript see: http://doi.org/10.7303/syn25992404 . Full results data sets can be found in the Supplement. Due to containing identifiable information, genotype data is available through controlled access via the corresponding authors following successful access to dbGaP dataset phs000979

Field-specific reporting

Please select the one below that is the best fit for your research. If you are not sure, read the appropriate sections before making your selection.

[x] Life sciences [ ] Behavioural & social sciences [ ] Ecological, evolutionary & environmental sciences

For a reference copy of the document with all sections, see nature.com/documents/nr-reporting-summary-flat.pdf

Life sciences study design

All studies must disclose on these points even when the disclosure is negative.

Sample size	Sample sizes were determined by availability of DNA samples of case and control status from each brain region
Data exclusions	11 samples from DLPFC were excluded from analysis due to having a strong, unexplained source of variance. This is described in the manuscript. They were excluded from all experiments after QC.
Replication	We performed secondary sensitivity analyses within each brain region after stratifying by ethnicity/ancestry, and by case/control status. We also performed comparisons across the two brain regions under study as a form of replication/validation. Results of all of these analyses are stated in the paper. Overall our results were very replicable across data subsets.
Randomization	N/A - these were case-control postmortem human studies; samples were collected from medical examiners offices and were retrospective
Blinding	N/A - these were case-control postmortem human studies. Groups were biologically defined by clinical or genetic data, the researchers had no role in grouping

Reporting for specific materials, systems and methods

We require information from authors about some types of materials, experimental systems and methods used in many studies. Here, indicate whether each material, system or method listed is relevant to your study. If you are not sure if a list item applies to your research, read the appropriate section before selecting a response.

Materials & experimental systems

n/a	Involved in the study	
x		Antibodies
x		Eukaryotic cell lines
x		Palaeontology and archaeology
x		Animals and other organisms
	x	Human research participants
x		Clinical data
x		Dual use research of concern

Methods

n/a	Involved in the study	
x		ChIP-seq
x		Flow cytometry
x		MRI-based neuroimaging

Human research participants

Policy information about studies involving human research participants

Population characteristics	Of the 183 unique donors included in this study, there were 117 males and 66 females between the ages of 18 and 96. 121 self reported as African American, and 62 self reported as Caucasian. 106 were neurotypical controls, and 77 had a schizophrenia diagnosis.
Recruitment	Human tissue samples were collected from medical examiners offices based on manner of death. Previous analyses by our group explored potential biases here: https://journals.plos.org/plosone/article?id=10.1371/journal.pone.0222565. "Donors did not differed significantly from non-donors on any demographic or clinical factors ascertained, including age, sex, diagnosis of the donor, or in the relationship of the next-of-kin being contacted (p>0.05).
Ethics oversight	Brain donation protocols were approved by IRB and oversight bodies.Legal next-of-kin gave informed consent to brain donation according to protocols Maryland Department of Health MDHMH# 12-24 (MD), National Institute of Mental Health [NIMH]# 90-M-014 (for brain donations in DC and VA), and Western Institutional Review Board [WIRB] # 1126332 (for brain donations in MD, WMU, UND), respectively. Subsequent research on human postmortem brain tissue - including the whole genome bisulfite sequencing performed here - is not classified as Human Subjects Research (since postmortem subjects are not classified as Human Subjects, per the Department of Health and Human Services in DHHS 45 CFR §46.102(e)(1):).

Note that full information on the approval of the study protocol must also be provided in the manuscript.
